# Supplementary material for: Health care workers intention to accept COVID-19 vaccine and associated factors in southwestern Ethiopia, 2021
Source: PLoS One. 2021 Sep 3;16(9):e0257109. doi: 10.1371/journal.pone.0257109 (PMC8415602; doi:10.1371/journal.pone.0257109)
Supplement: S1 Questionnaire — (DOCX) [file pone.0257109.s001.docx]

Annex

INTENTION TO ACCEPT COVID-19 VACCINE QUESTIONNAIRE

Dear Sir/Madam

Mr. Abiy Tadesse, Mr. Daniel Shiferaw and Mr. Aklilu Mamo are conducting research on “Health Care Workers Intention to Accept COVID-19 Vaccine and Associated Factors in Southwestern Ethiopia, 2021”. The finding from this study will help to improve the COVID-19 vaccine acceptance in healthcare workers which will battle the pandemics of COVID-19. Therefore, we kindly request your truthful response to this self-administered questionnaire. Your participation in this study is voluntary, but your contribution is helpful in this critical time. The study may consume your time and your response will be completely confidential. If you have any questions regarding this research please feel free to contact the principal investigators by the following addresses (Abiy Tadesse: +251917461988, Daniel Shiferaw: +251931975151, Aklilu Mamo: +251961933071).

Socio-demographic characteristics of the participants

| SNO | Name of data collector | _____________________________ |
| --- | --- | --- |
|  | Code of the questionnaire | _____________________________ |
| 101 | Age | _______________years |
| 102 | Sex | 1. Male 2. Female |
| 103 | Marital Status | 1. Single 2. Married 3. Widowed 4. Divorced |
| 104 | Professional categories | 1. Physicians 2. Nurse 3. Midwifery 4. Medical laboratory 5. Pharmacist |
|  |  | 1. Radiology technicians 2. Psychiatry professionals |
| 105 | Highest qualification level | 1. Diploma 2. Degree 3. Masters |
| 106 | Monthly salary in USD | 1. 68.4 – 91.2 2. 91.3 – 182.4 3. >182.4 |
| 107 | Number of families in home | 1. 1 2. 2 3. 3-4 4. 5-6 5. >7 |

Health status and COVID-19 experience

| SNO | Questions | Responses |
| --- | --- | --- |
| 201 | Do you have history of chronic illness? | 1. Yes 2. No |
| 202 | Do you have any of the following diseases? (Type 2 diabetes mellitus, Chronic Obstructive Pulmonary Disease (COPD), Cancer, Kidney Failure, Heart diseases, Sickle Cell Anemia) | 1. Yes 2. No |
| 203 | Do you have any of the following diseases? (Type 1 diabetes mellitus, Hypertension, Bone marrow transplant, Cerebrovascular diseases or stroke, Cystic Fibrosis, Asthma, Taking steroids or immunosuppressant drugs, Hepatic diseases, Thalassemia, Lung fibrosis) | 1. Yes 2. No |
| 204 | How do you perceive your risk for COVID-19? | 1. High 2. Medium 3. Low |
| 205 | Do you have history of COVID-19 infection? | 1. Yes 2. No |
| 206 | Do you know any friends, neighbors ,or colleagues infected by Coronavirus | 1. Yes 2. No |

COVID-19 related knowledge questions

| SNO | Items | Responses |
| --- | --- | --- |
| 301 | A suspected case is a patient with acute respiratory illness and recent history of travel to covid-19 risk area. | 1. True 2. False 3. I don’t know |
| 302 | A person with laboratory confirmation of COVID 19 infection, irrespective of clinical signs and symptoms is a confirmed case. | 1. True 2. False 3. I don’t know |
| 303 | A suspected case is any patient with fever and at least cough or shortness of breath. | 1. True 2. False 3. I don’t know |
| 304 | Any patient with a history of contact with a confirmed or probable COVID 19 case in the last 14 days before symptom onset is a suspected case. | 1. True 2. False 3. I don’t know |
| 305 | The main clinical symptoms of COVID-19 are fever, fatigue, dry cough, and myalgia. | 1. True 2. False 3. I don’t know |
| 306 | Unlike symptoms of common cold, stuffy nose, running nose, and sneezing are less common in persons infected with the SARS-COV-2. | 1. True 2. False 3. I don’t know |
| 307 | Eating Monkey, Bat or contacting wild animals would result in the infection by the SARS-COV-2. | 1. True 2. False 3. I don’t know |
| 308 | Patients with COVID-19 cannot spread the virus to others when they do not show signs and symptoms of the disease. | 1. True 2. False 3. I don’t know |
| 309 | The SARS-COV-2 virus spreads via respiratory droplets of infected individual. | 1. True 2. False 3. I don’t know |
| 310 | The incubation period of COVID-19 lasts up to 14days. | 1. True 2. False 3. I don’t know |
| 311 | Children and young adults are less likely to be infected with COVID 19 thus, precautionary measures are not necessary to prevent the infection. | 1. True 2. False 3. I don’t know |
| 312 | Not all patients infected with COVID-19 will develop severe cases. | 1. True 2. False 3. I don’t know |
| 313 | Patients with underlying chronic disease conditions are at higher risk of infection and death from COVID 19. | 1. True 2. False 3. I don’t know |
| 314 | Avoiding handshakes, crowded places, and public transportation could help to prevent COVID-19 | 1. True 2. False 3. I don’t know |
| 315 | Antibiotics are the first line of treatment when you suspect or have a confirmed case of COVID-19. | 1. True 2. False 3. I don’t know |
| 316 | Early recognition and supportive treatment help most patients recover from the infection since there is no effective cure for COVID-19. | 1. True 2. False 3. I don’t know |
| 317 | Isolation and treatment of people who are infected with the COVID-19 virus are effective ways to break the chain of transmission | 1. True 2. False 3. I don’t know |
| 318 | Personal protective equipment (PPE) like respiratory protection cannot effectively protect the users if it is not properly and consistently worn. | 1. True 2. False 3. I don’t know |
| 319 | Wearing face masks are used to protect both the Health care Workers and the patient. | 1. True 2. False 3. I don’t know |

Attitude towards COVID-19 preventive measures

| 401 | Do you believe that social distancing and hand washing could prevent COVID-19? | 1. Yes 2. No 3. Not sure |
| --- | --- | --- |
| 402 | Do you have confidence in the current preventive measures put in place by the Government to mitigate COVID 19? | 1. Yes 2. No 3. Not sure |
| 403 | Have you attended any social events recently? | 1. Yes 2. No 3. Not sure |
| 404 | Are you confident to provide care to a suspected case of COVID 19? | 1. Yes 2. No 3. Not sure |
| 405 | Do you believe that the COVID-9 vaccine can prevent infection? | 1. Yes 2. No 3. Not sure |
| 406 | Health insurance or incentives can motivate health care workers directly involved in the management of COVID 19 patients? | 1. Yes 2. No 3. Not sure |
| 407 | Are you ready to participate in community sensitization on COVID 19? | 1. Yes 2. No 3. Not sure |

COVID-19 prevention practices

| SNO | Items | Responses |
| --- | --- | --- |
| 501 | Do you wash your hands or sanitize your hands regularly? | 1. Yes 2. No |
| 502 | Do you regularly use facemask at point of care (when rendering service to sick patients)? | 1. Yes 2. No |
| 503 | Do you use a facemask when you have flu-like symptoms? | 1. Yes 2. No |
| 504 | Do you use non-conventional remedies (Honey, garlic, ginger, and lime) when you have flu-like symptoms? | 1. Yes 2. No |
| 505 | In recent times, have you worn a face mask when leaving your home? | 1. Yes 2. No |

Vaccine hesitancy questions

| SNO | Items | Responses |
| --- | --- | --- |
| 601 | Have you ever refused a vaccine for yourself or a child because you considered it useless or dangerous? | 1. Yes 2. No |
| 602 | Have you ever postponed a vaccine recommended by a physician? | 1. Yes 2. No |
| 603 | Have you ever had a vaccine for a child or yourself despite doubts about its efficacy? | 1. Yes 2. No |

Intention to accept COVID-19 vaccine

| SNO | Item | Responses |
| --- | --- | --- |
| 701 | Will you get the COVID-19 vaccine if it is available? | 1. Yes 2. No |

Thank you very much for your information and time!
