# Supplementary material for: Health care workers intention to accept COVID-19 vaccine and associated factors in southwestern Ethiopia, 2021
Source: PLoS One. 2021 Sep 3;16(9):e0257109. doi: 10.1371/journal.pone.0257109 (PMC8415602; doi:10.1371/journal.pone.0257109)
Supplement: Attachment — Submitted filename: Response to editor and reviewers.docx [file pone.0257109.s002.docx]

**Response to editor**

**We would like to extend our thanks for valuable comments from the editor.**

1. Please ensure that your manuscript meets PLOS ONE's style requirements, including those for file naming.

- **We have ensured that our manuscript is written in the journal’s style requirements, including for file naming.**

2. Please provide additional details regarding participant consent. In the ethics statement in the Methods and online submission information, please ensure that you have specified what type you obtained (for instance, written or verbal, and if verbal, how it was documented and witnessed). If your study included minors, state whether you obtained consent from parents or guardians. If the need for consent was waived by the ethics committee, please include this information. Once you have amended this/these statement(s) in the Methods section of the manuscript, please add the same text to the “Ethics Statement” field of the submission form (via “Edit Submission”).

- We have added additional details regarding participant consent in revised manuscript. Our study doesn’t include minors and we have added “ethics statement” during the submission of the revised manuscript.

3. Please include additional information regarding the tool or questionnaire used in the study and ensure that you have provided sufficient details that others could replicate the analyses. For instance, if you developed the tool or questionnaire as part of this study and it is not under a copyright more restrictive than CC-BY, please include a copy, in both the original language and English, as Supporting Information. If the questionnaire is published, please provide a citation to the (1) questionnaire and/or (2) original publication associated with the questionnaire.

- We have uploaded questionnaire as supporting information.

4. We note that you have indicated that data from this study are available upon request. PLOS only allows data to be available upon request if there are legal or ethical restrictions on sharing data publicly. For information on unacceptable data access restrictions, please see <http://journals.plos.org/plosone/s/data-availability#loc-unacceptable-data-access-restrictions>.

- The data underlying the results contain the potential identification of our study participants and have some ethical restrictions as set by the ethical review committee of Mizan Tepi University, College of Health Sciences. However, the row datasets will be available on reasonable request after requesting [abiyutad@mtu.edu.et](mailto:abiyutad@mtu.edu.et) .

**Comments to reviewers**

Reviewer #1

1. The conclusion repeated some the results but did not provide new insights or ideas on the topic addressed.

- Thank you reviewer for this important observation. We have made conclusion based on your comments in the revised manuscript.

2. Introduction Page 10, line 3: Please, correct the sentence “...cases outside a China”

- We have corrected the sentences with in the revised manuscript.

3. Page 11, line 8-10 (last paragraph of ‘introduction’ section): Please, make clear the aouthor’s hipothesis.

- Good observation dear reviewer. We have corrected it accordingly.

4. What abouth the ethics? Please, provide more informations. Did the participants sign the free informed consent?

- Thank you reviewer for such comments, and we have provided more information like the form of informed consent in the revised manuscript.

5. Page 12, ‘Sample Size and Sampling Technique’ section: How do the authors estimated the vaccine acceptance in 50%? Please, provide the reference for this assumption

- We used the proportion of vaccine acceptance in professionals as 50% because; during sample size estimation we didn’t find any study that identified the proportion of COVID-19 vaccine acceptance in Ethiopia. Therefore we used a proportion of vaccine acceptance of 50 % which could provide the minimum sample size for our study.

6. Page 13, lines 2-3: The sentence “The lists of all health care workers were obtained from both hospitals, and the lottery method was used to select 261 samples from MTUTH and 162 from GTSGH.” is redundant. Please, remove it.

- Thank you for good observation, and we have removed the redundant sentence.

7. ‘Socio-demographic characteristics of the respondents” section and table 1: Please, present the participant’s monthly salary in US dollars. It is more informative.

- We have changed the monthly salary of professionals stated in Ethiopian Birr to USD.

8. It would be interesting to deepen the discussion about if (and how) socio-cultural characteristics of the population could be related to the hesitancy to take the COVID-19 vaccine. In addition, insights on how to overcome this hesitancy should be proposed at the end of the discussion or in the conclusion.

- Thank you reviewer for important idea but identifying the association between socio-cultural characteristics and vaccine hesitancy was not objective of the study. Dear reviewer we have added the insights on how to overcome vaccine hesitancy in the conclusion.

9. In addition, some formatting errors, such as parentheses and capital letters, need to be corrected

- We have edited our manuscript in revised manuscript.

**Reviewer # 2**

1. It would be nice to have more data regarding the current situation in Ethiopia and the situation around the time of the study. Had cases continued to rise at that time? How bad was the area surveyed hit?

- Thank you reviewer for important observation. We have incorporated this comment with in the revised manuscript. You can see under introduction part starting from line 8.

2. Are there any data regarding public immunization willingness in Ethiopia?

- Thank you dear reviewer for important insight. We have included the data regarding public willingness to COVID-19 vaccine in main manuscript in the introduction section. The findings regarding public willingness showed that willingness is low.

3. How well did the residents of Ethiopia accept public health recommendations for things like masks etc? This would help better frame the research.

- We have incorporated this comment with in revised manuscript. Dear reviewer you can get this in line 28 of introduction part.

4. Also- what is the current vaccine avaialbility in Ethiopia? Are any vaccine available?

- We have incorporated this with in revised manuscript. It is located in the last paragraph of the introduction part.

5. General: Recommend better editing for English throughout.

- Thank you dear reviewer for good recommendation. We have edited the revised manuscript.

6. COVID-19 should be capitalized throughout

- We have corrected it accordingly.

7. Abstract: Methods should be clear that this is a survey of healthcare workers at two hospitals.

- Tank you dear reviewer important insight. Our study is facility based cross-sectional study and it is not survey. The study was conducted in two hospitals and participants were selected randomly by lottery methods after proportional allocation to each hospitals based on their professional numbers.

8. How was the survey conducted? Was it anonymous? Was it a paper survey or administered in person?

- Thank you dear reviewer for interesting questions. Data were collected by self-administered questionnaire. First participants were selected randomly by lottery method in both hospitals and data collectors provided questionaries’ to selected professionals.

9. What types of healthcare workers were eligible? Beyond Nurses, what other types of healthcare workers were eligible to be included? Please also give more information on what "first degree holders" are.

- Thank you dear reviewer for important observations. Health care workers who involved in health care and direct contact with patients were eligible for the study. Beyond nurses all professionals including physician, midwifery, medical laboratory technologist, pharmacist, radiology technicians and psychiatry professionals were eligible and included within the study. First degree holder in our study is to mean those professional who have bachelors of Science degree (First degree) in any field of health like medicine, nursing, midwifery or others field.

10. Given that "Others" are the reference population, we need more information about that others" actually are. Consider making nurses or a different, larger, group the reference group.

- Thank you dear reviewer for important insight. Others included radiology technicians and psychiatry professionals. We have changed the reference category based on your recommendation and in the revised manuscript we put nurses as a reference group.

Reviewer # 3

1. The authors are suggested to give more details on the context/ development of COVID-19 in (Southern) Ethiopia near the data collection period (March 2021), which could influence HCW’s acceptance of the vaccine.

- Thank you dear reviewer for good observation. We have included the situation of COVID -19 in Ethiopia in the revised manuscript. It is located within the introduction part starting from line 7 of introduction part.

2. The scoring of COVID-19 attitude seemed problematic. I could not understand why the responses were ranked from positive (3 points), negative (2 points) to not sure (1 point). Should “not sure” score 2 points while negative 1 point? Furthermore, since there were 7 items, the total scores should range from 7 to 21, not 7 to 18. Can the authors show the internal consistency of these 7 items? In the Methods, please also make it clear that these measures are referring to attitude toward COVID-19 preventive measures.

- We appreciate dear reviewer for important observation. It was mistakenly written with in old version of the manuscript and your insight was correct. During analysis of the result, attitude responses was scored as follows (yes = 1, no = 0 and not sure = 0). And responses to reversed questions were reversed when assigning the points (Yes=0, not sure = 0 and No=1). As stated within the result, the attitude score ranged from 0 to 7. This scoring is consistent with previous study conducted in Healthcare Workers’ Attitude toward COVID-19. Dear reviewer this is link to access the previous study (https://doi.org/10.2147/JMDH.S287156). The mean attitude score (4.9 ±1.6) was computed and participants' were considered as having a positive attitude if the attitude score ≥ mean attitude score. We have corrected the scoring of attitude items which was consistent with our result of attitude responses within revised manuscript. We have also included statements referring to attitude toward COVID-19 preventive measures in method parts.
- The internal consistency of 7 attitude items was 0.818 and we have uploaded the SPSS output of reliability below.

3. Responses to the question of COVID-19 vaccine hesitancy were dichotomised to “yes” and “no”. Can the authors presented the original scale of the responses?

- Thank you reviewer for important observation. We have evaluated self-reported vaccine hesitancy of the participants according to the WHO definition using previously adapted three questions. We have classified participants’ response to vaccine hesitancy to yes and no which is consistent to previous study (.DOI:https://doi.org/10.1016/j.jhin.2020.11.020).

4. The logistic regression models used in the study appeared to be “multivariable” (1 dependent variable, multiple independent variables) instead of “multivariate” (multiple dependent variables). Please correct.

- Thank you for important comment. We have changed it in the revised manuscript.

5. In the abstract the sample size was 423, whereas in the main text it was 405. Please correct.

- We have corrected it with in the abstract part of the revised manuscript.

6. Table 2: Was there any particular reason to divide the chronic diseases into the two categories listed?

- Thank you dear reviewer for important observation. We categorized chronic disease that professionals’ had for description purpose.

7. Table 4: Please indicates the correct answers for the questions on COVID-19 knowledge.

- Thank you for good review. We have presented the correct knowledge responses (**bold**) below in the table.

| Variables | Category |
| --- | --- |
| A suspected case is a patient with acute respiratory illness and recent history of travel | **True** |
|  | False |
|  | I don’t know |
| A person with laboratory confirmation of COVID 19 infection, irrespective of clinical signs and symptoms is a confirmed case | **True** |
|  | False |
|  | I don’t know |
| A suspected case is any patient with fever and at least cough or shortness of breath | **True** |
|  | False |
|  | I don’t know |
| Any patient with a history of contact with a confirmed or probable COVID 19 case in the last 14 days before symptom onset is a suspected case | **True** |
|  | False |
|  | I don’t know |
| The main clinical symptoms of COVID-19 are fever, fatigue, dry cough, and myalgia | **True** |
|  | False |
|  | I don’t know |
| Unlike symptoms of common cold, stuffy nose, running nose, and sneezing are less common in persons infected with the SARS-COV-2 | **True** |
|  | False |
|  | I don’t know |
| Eating Monkey, Bat or contacting wild animals would result in the infection by the SARS-COV-2 | **True** |
|  | False |
|  | I don’t know |
| Patients with COVID-19 cannot spread the virus to others when they do not show signs and symptoms of the disease | True |
|  | **False** |
|  | I don’t know |
| The SARS-COV-2 virus spreads via respiratory droplets of infected individual | **True** |
|  | False |
|  | I don’t know |
| The incubation period of COVID-19 lasts up to 14days | **True** |
|  | False |
|  | I don’t know |
| Children and young adults are less likely to be infected with COVID 19 thus, precautionary measures are not necessary to prevent the infection | True |
|  | **False** |
|  | I don’t know |
| Not all patients infected with COVID-19 will develop severe cases | **True** |
|  | False |
|  | I don’t know |
| Patients with underlying chronic disease conditions are at higher risk of infection and death from COVID 19 | **True** |
|  | False |
|  | I don’t know |
| Avoiding handshakes, crowded places, and public transportation could help to prevent COVID-19 | **True** |
|  | False |
|  | I don’t know |
| Antibiotics are the first line of treatment when you suspect or have a confirmed case of COVID-19 | True |
|  | **False** |
|  | I don’t know |
| Early recognition and supportive treatment help most patients recover from the infection since there is no effective cure for COVID-19 | **True** |
|  | False |
|  | I don’t know |
| Isolation and treatment of people who are infected with the COVID-19 virus are effective ways to break the chain of transmission | **True** |
|  | False |
|  | I don’t know |
| Personal protective equipment (PPE) like respiratory protection cannot effectively protect the users if it is not properly and consistently worn | **True** |
|  | False |
|  | I don’t know |
| Wearing face masks are used to protect both the Health care Workers and the patient | **True** |
|  | False |
|  | I don’t know |

8. Table 5: I have doubts on the question “Do you use non-conventional remedies (Honey, garlic, ginger, and lime) when you have flu-like symptoms?” as a measure of COVID-19 prevention practice. This dose not seem a proven/ recommended practice to prevent COVID-19.

- Good observation. These **none-conventional** remedies may increase the immunity of individuals in fighting the infection. The question is consistent with previous published literature (<https://doi.org/10.2147/JMDH.S287156>).

9. Please shows the overall prevalence with 95% CI of COVID-19 vaccine acceptance in the results and in the abstract.

- Thanks for important observation. We have included the 95% CI of COVID-19 vaccine acceptance in abstract and result.

10. Table 7: The percentages shown appeared to be cell percentages (rows and columns add up to 100%), which are not useful nor straightforward to interpret. The authors should use row percentages (percentages in the same row add up to 100%) to show the prevalence of vaccine acceptance in each subgroup of variables. Also, it seems more meaningful to use “Nurse” as the reference group instead of “Others” for the variable “Type of Profession”. Please also show the association of vaccine hesitancy and COVID-19 vaccine acceptance (not presented in the table) and which variables were adjusted in the adjusted models.

- Thank you dear reviewer for important observation. We have corrected the percentage of variables that’s sum to be give 100 %. We have also put nurse as reference category and association between vaccine hesitancy and COVID -19 vaccine acceptance.

11. Discussion 3rd paragraph: It is surprising to see that vaccine hesitancy was not associated with COVID-19 vaccine acceptance, since many prior studies have shown vaccine hesitancy/ previous vaccination history to be strongly associated with vaccine acceptance. I could not interpret the results because they were not presented (see comment 10).

- We have corrected this in our revised manuscript.

12. The vaccine acceptance among nurses appeared to rather low relative to other healthcare workers (~45% vs 54% based on the numbers presented in table 7). This is concerning because nurses constitute the largest health care workforce and have frequent contacts with patients. Can the authors discuss the finding?

- We are thankful for important observation. We have discussed this in the revised manuscript under discussion part.

13. The authors should elaborate the implications of their findings/ How their findings could be used to improve COVID-19 vaccine acceptance?

- We have incorporated this in the introduction and conclusion part of the revised manuscript.

14. The authors should discuss the limitations of their findings, which are absent in the article.

- We thanks for important recommendation. We have added this within revised manuscript.

15. The article would be much benefited from English editing.

- We have corrected this in our revised manuscript.
