# Supplementary material for: Health care workers intention to accept COVID-19 vaccine and associated factors in southwestern Ethiopia, 2021
Source: PLoS One. 2021 Sep 3;16(9):e0257109. doi: 10.1371/journal.pone.0257109 (PMC8415602; doi:10.1371/journal.pone.0257109)
Supplement: Attachment — Submitted filename: Response to editor and reviwers.docx [file pone.0257109.s003.docx]

**Response to editor**

**We would like to extend our thanks for valuable comments from the editor.**

1. Please review your reference list to ensure that it is complete and correct. If you have cited papers that have been retracted, please include the rationale for doing so in the manuscript text, or remove these references and replace them with relevant current references. Any changes to the reference list should be mentioned in the rebuttal letter that accompanies your revised manuscript. If you need to cite a retracted article, indicate the article’s retracted status in the References list and also include a citation and full reference for the retraction notice.

- **We have ensured that our reference list is complete and correct.**

**Comments to reviewers**

**Reviewer # 2**

1. The authors claim it is not a two hospital survey but then the methods explicity state the study was conducted in MTUTH and GTSGH- two hteaching hospitals. They randomly selected these two hospitals out of a total of four in the southwestern part of ethiopia, but it still was only two hospitals. Thus, this is a fairly limited study given that it only explores attitudes in two teaching hospitals. This should be described as a limitation.

- Thank you reviewer for this important observation. We have included this in limitation part of the revised manuscript.

2. I'm confused still on how the sutvey was administered. "self-administered" isn't very descriptive. Was it a paper survey or on a computer? Were responses anonymous?

- Thank you reviewer for an interesting question. The study was paper survey and anonymous. The name of the participants was not included in the paper. No person identifier information was included in the paper. We have included this in Data Collection Tool, Quality Control, and Procedure and Ethical Considerations parts of the revised manuscript.

3. More detail on the type of healthcare workers should be included in the main manuscript.

- Thank you for important observation. We have added the types of health care worker included in the study in inclusion part of the revised manuscript.

4. Finally, there are a lot of tables. I suggest condensing these down

- Thank you for such important suggestion. But as journal requirement there is no limitation for numbers of table. And for more clarity of the finding we prefer to present by table as many of questions we used are long.

**Reviewer #3**

1. Please include the internal consistency results of the 7 items on attitudes toward COVID-19 preventive measures in the methods.

- Thank you dear reviewer for important insight. We have included for this in the methods parts of the revised manuscript.

2. In the abstract, some results (e.g., AOR=15.18) seem not consistent with those presented in the table 7. Please also indicate the reference group for physicians.

- We appreciate for such vision. We have corrected it in the abstract part of the revised manuscript. The reference group was nurses.

3. Please indicate which item is reversed code in Table 4.

- Thank you dear reviewer for such comment. We have indicated for reversed item during scoring in the table 4 of the revised manuscript by symbol ***.**
